# Gastrointestinal involvement in juvenile systemic sclerosis: development of recommendations for screening and investigation

**DOI:** 10.1186/1546-0096-12-S1-P52

**Published:** 2014-09-17

**Authors:** Clare Pain, Tamas Constantin, Eilleen Baildem, Christian Beyer, Henning Lenhartz, Michael Blackley, Dana Nemcova, Clarissa Pilkington, Ivan Foeldvari

**Affiliations:** 1 Pediatric Rheumatology, Liverpool, UK; 2 Pediatric Rheumatology, Budapest, Hungary; 3 Pediatric Cardiology, Hamburg, Germany; 4 Pediatric Gastroenterology, Hamburg, Germany; 5 Pediatric Rheumatology, Indiana, USA; 6 Pediatric Rheumatology, Prague, Czech Republic; 7 Pediatric Rheumatology, London, UK; 8 Pediatric Rheumatology, Hamburg, Germany

## Introduction

There are currently no agreed recommendations on how to investigate children for gastrointestinal (GI) involvement in Juvenile Systemic Sclerosis (JSSc). The aim of screening is to detect disease early to facilitate early aggressive therapy and improve outcomes. GI involvement at diagnosis incurs a worse outcome [1]. Most deaths occur early in the disease course [1, 2].

## Objectives

To develop recommendations for investigation of GI involvement in JSSc, based on paediatric evidence and where this was lacking, consensus expert agreement.

## Methods

Members of the PRES Scleroderma Working Group were invited to participate; additionally a paediatric gastroenterologist was invited. A nominal group technique was used. 75% consensus was defined as agreement.

## Results

Table 1 shows the recommendations for screening for GI involvement at baseline and at defined time points from diagnosis. Other recommendations agreed by the group which are relevant at any stage in the disease course are as follows:

**Table 1:** 

**Gastrointestinal**	**Baseline**	
	
	All patients should have a barium swallow to assess for dysmotility or stricture and 24 hour pH monitoring for GORD and progress to upper GI endoscopy if any abnormality detected	
	
	**Follow-up**	
	
	Every 3 years or sooner if worsening lung involvement and/or worsening GI symptoms	Upper GI endoscopy Barium swallow 24 hours pH monitoring

*screening guidelines are based on asymptomatic patients. However, children may need more frequent monitoring depending on clinical status and abnormalities detected on previous investigation.

## Abbreviations

BP: blood pressure; ECG: electrocardiogram; ECHO: echocardiogram; MRI: magnetic resonance imaging; HRCT: high resolution computerised tomography; PFT: DLCO pulmonary function tests with diffusion capacity of lung for carbon monoxide; 6MWT: 6 minute walk test; NT BNP: *N-terminal pro-brain natriuretic peptide; GORD: gastro-oesophageal reflux disease*.

## Conclusion

JSSc has a significant mortality particularly early on in the disease course. The objective of an aggressive screening program is to identify GI involvement at a stage which may be amenable to treatment. The recommendations developed by this group aim to standardise care and improve outcomes in this rare disease.

## Disclosure of interest

None declared.

